# Mechanical Behavior and Crack Evolution of Goaf Surrounding Rock with Different Roof-Contacted Filling Rates

**DOI:** 10.3390/ma16124435

**Published:** 2023-06-16

**Authors:** Jie Wang, Kaifei Huang, Jianxin Fu, Weidong Song

**Affiliations:** 1 School of Civil and Resources Engineering, University of Science and Technology Beijing, Beijing 100083, China; 18810582761@163.com (J.W.); songwd@ustb.edu.cn (W.S.); 2 State Key Laboratory of High-Efficient Mining and Safety of Metal Mines of Ministry of Education, University of Science and Technology Beijing, Beijing 100083, China

**Keywords:** acoustic emission, crack evolution, biaxial loading, stress concentration

## Abstract

The goaf formed by mining is filled and treated, which greatly improves the safety and stability of the surrounding rock. During the filling process, the roof-contacted filling rates (RCFR) of goaf were closely related to the stability control of the surrounding rock. The influence of the roof-contacted filling rate on the mechanical characteristics and crack propagation of the goaf surrounding rock (GSR) has been studied. Biaxial compression experiments and numerical simulation experiments were conducted on samples under different operating conditions. The results were as follows: (1) The peak stress, peak strain, and elastic modulus of the GSR are closely related to the RCFR and the goaf size; they increase with the increase of the RCFR, and decrease with the increase of the goaf size; (2) In the initial loading stage, a small number of cracks are generated, and the acoustic emission ringing count increases slowly. The mid-loading stage is the crack initiation and rapid expansion, and the cumulative ring count curve shows a “stepwise” growth. In the later loading stage, cracks continue to propagate and form macroscopic fractures, but the number of rings significantly decreases; (3) Shear cracks are prone to occur in the rock part of the GSR; tensile cracks are prone to occur in the backfill; and the crack propagation speed in the rock is faster than in the backfill. Stress concentration is the direct cause of GSR failure. The maximum concentrated stress of rock mass and backfill is 1~2.5 times and 0.17~0.7 times of the peak stress of the GSR, respectively.

## 1. Introduction

During underground mining, a large number of complex goafs are generated. Under the action of high ground stress, surrounding rock deformation and failure, rock burst, collapse, roof fall, and other disasters are easily caused [1,2,3,4,5]. In order to ensure mining safety, control the ground pressure in the goaf, and reduce the occurrence of disasters, most mines deal with the goaf through filling treatment [6,7,8]. This has led to a large number of scholars studying the preparation process, material selection, and stratification state of filling materials [9,10,11,12,13,14].

With the widespread application of the backfill mining method in mines, composite support structures composed of backfill and rock mass are becoming increasingly common in mines. This structure has been extensively studied by many scholars and has achieved fruitful results [15,16,17,18]. The pressure mechanism of composite models has been studied by some scholars. Tan et al. [19] conducted mechanical experiments on composite specimens wrapped in surrounding rock, exploring the mode and mechanism of the composite pressure bearing effect between the backfill and the surrounding rock. Song et al. [20,21] studied the interaction between rock mass and backfill as a system, participating in the support function together. They believe that the increase in filler strength gradually increases the peak strength, residual strength, and axial strain during the failure period of the composite material model, and during the loading process, the composite material model transitions from tensile failure to shear failure. The interface between rocks and filling materials has been studied by some scholars. Fang, K et al. [22,23,24] studied the effects of curing temperature, sulfate, curing stress, drainage conditions, and filling rate on the shear behavior of the interface between cemented backfill and rock. Regarding the damage and failure mechanism of the composite model, Wang et al. [25] studied and analyzed the mechanical properties and crack spatial distribution of GSR, and established a damage constitutive model of the sample. It can be seen that the above scholars’ experiments were all based on considering the complete filling of the goaf with filling slurry. However, in reality, the goaf cannot be fully filled with filling slurry during the filling process, so it will retain a portion of the unconnected space.

The reasonable use of numerical simulation can help compensate for the shortcomings of indoor experiments, such as using PFC programs for simulation analysis, where the generation and evolution of cracks in the sample can be clearly observed. This software has been used by many scholars to analyze the failure mechanism of samples. Zhao et al. [26] used the PFC2D program to simulate the damage evolution of a single fractured rock mass under high strain rates, and the crack propagation forms were classified. Manouchehrian et al. [27,28] conducted a servo test simulation; the crack initiation and propagation process of rock samples under different confining pressures as well as the changes in the displacement field were analyzed from a microscopic perspective. The failure characteristics of specimens under deteriorating conditions have been studied by some scholars through pre-fabricated cracks. Liu et al. [29] proposed an improved fluid flow model for fractured porous media and founded that initial defects in the same direction may lead to a well-connected crack network with high global efficiency. On the shape of the fissure, Haeri et al. [30] also used PFC2D to simulate the L-shaped non-persistent fractured rock mass. From the research of the above scholars, it can be seen that the use of PFC programs has significant advantages in the study of cracks.

On the basis of understanding that the goaf cannot be filled with backfill, the GSR samples in this article were prepared, and the effects of RCFR and goaf size on various aspects of GSR samples were studied. The GSR sample was subjected to biaxial loading experiments, and its mechanical properties, failure modes, and acoustic emission characteristics were analyzed. Numerical simulation experiments were also conducted using the PFC program, revealing the crack evolution and failure mechanism of GSR specimens, and exploring the characteristics of stress concentration. Through this study, the locations where stress concentration and failure are prone to occur in the combined model under different filling conditions and top contact rates have been identified. The precise protection and secondary support foundation of the mine after filling ensures the safe and stable mining of the mine, but it also provides a reference basis for subsequent scholars to study the combination structure of backfill and rock mass.

## 2. Materials and Methods

### 2.1. Test Method

In the process of filling and mining in mines, the “filling one mining one” model has usually been used. This article prepares GSR combination model samples based on the state of the surrounding rock and backfill combination after the excavation filling. Sandstone was processed into GSR samples in the laboratory. The size side length is 100 mm × 100 mm × 100 mm, and the side length of goaf is 20 mm × 20 mm × 100 mm, 30 mm × 30 mm × 100 mm, 40 mm × 40 mm × 100 mm.

**Figure 1:**
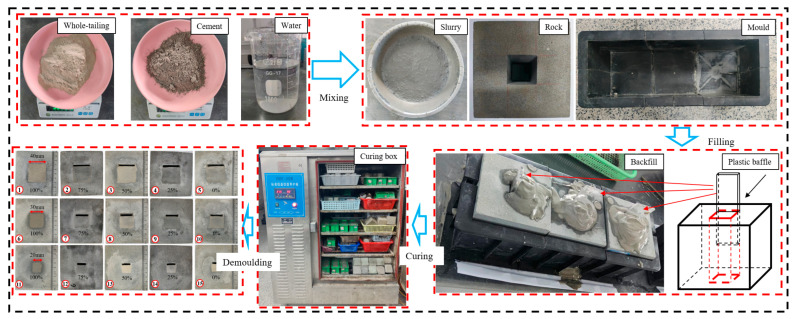
Preparation of GSR (1–15) all composite models.

To prepare the GSR with different RCFR conditions, plastic baffles were inserted into the goaf to control the RCFR. The prepared filling slurry has a cement-sand ratio of 1:6 and a solid mass concentration of 75%. The prepared GSR was placed in a curing box with a humidity of ≥90% and a temperature of 20° ± 2. The GSR demould after 2 days of curing, and then placed in the curing box for 26 days of curing for a total of 28 days. Finally, the biaxial loading experiment was carried out. The preparation process of the GSR is shown in Figure 1.

**Figure 2:**
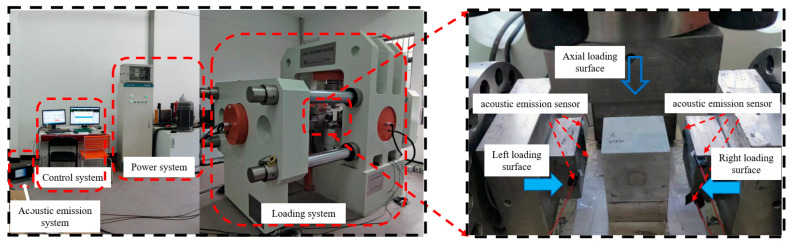
Experimental instrument and loading diagram.

This experiment was completed on the rock true triaxial dynamic and static load experimental system. The experimental steps are as follows: The GSR was placed on a fixed loading platform, and three acoustic emission sensors were added to the left and right surfaces. The sampling threshold of the acoustic emission test and analysis system was set to be 40 dB and the sampling frequency was 40 MHz. The left and right loading surfaces were set to 30 MPa confining pressure, and the axial loading surface was loaded at 0.002 mm/s until the sample was completely destroyed. The front and rear were free surfaces and were not loaded. The experiment systems are shown in Figure 2.

**Figure 3:**
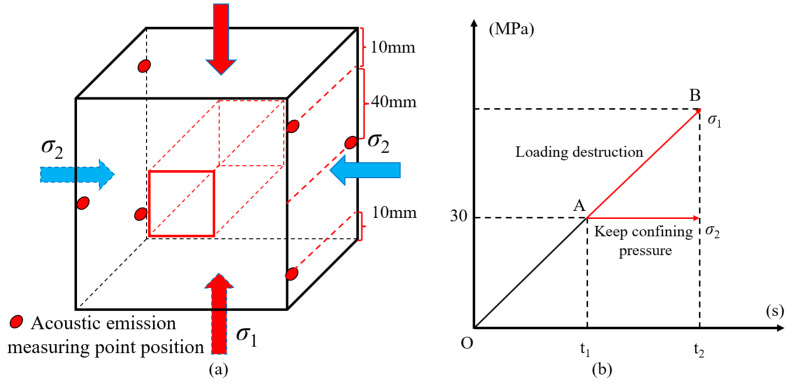
The loading mode of the GSR (**a**) loading direction; (**b**) path of loading; (A) *σ*_1_ = *σ*_2_ = 30 MPa; (B) Peak stress point.

The loading mode of the GSR in the biaxial loading experiment is shown in Figure 3a. Among them, the upper and lower sides were used as the loading surface, and three acoustic emission monitoring points were distributed on the left and right sides. The loading path of the experiment is shown in Figure 3b. Firstly, the *σ*_1_ and *σ*_2_ of the GSR were loaded to the predetermined pressure state (OA) at a loading rate of 0.2 MPa/s. At this time, *σ*_1_ = *σ*_2_ = 30 MPa, and then keep *σ*_2_ unchanged, controls the axial wall to maintain the rate of 0.002 mm/s to axially load the GSR until the axial stress of the model exceeds the ultimate bearing capacity at this time, and finally loses stability.

### 2.2. Numerical Simulation (PFC3D)

#### 2.2.1. Determination of Micro-Mechanical Parameters of the GSR

The advantage of PFC particle flow software is that it is not limited by deformation, and mechanical problems can be easily handled in discontinuous media. It has achieved good results in simulating materials such as rock masses, reflecting the different physical relationships of multiphase media, and simulating the phenomenon of crack separation in multiphase media. The real-time reflection of fracture process and results have advantages in studying medium fracture. Therefore, PFC3D particle flow software was used for this simulation experiment.

**Figure 4:**
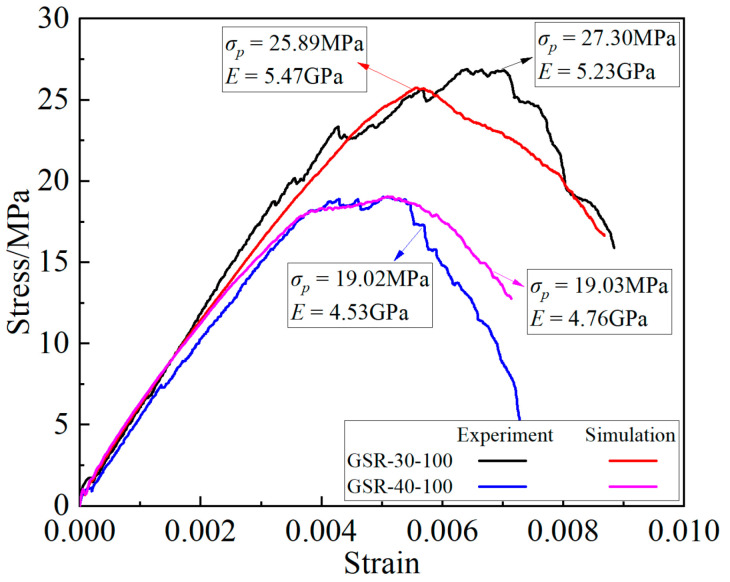
Comparison of deviatoric stress-strain curves GSR-30-100 and GSR-40-100.

The numerical simulation experiment plan is consistent with the indoor experiment, but ten sets of simulation experiments with goaf sizes of 25 mm and 35 mm were added. The “trial and error method” was used in this simulation experiment to calibrate the mechanical parameters of rocks and filling materials, as shown in Table 1. Based on the inherent characteristics of PFC3D software, the stress-strain curve of the O to t_1_ process cannot be effectively monitored. Therefore, the deviatoric stress-strain curve is obtained by monitoring the loading after t_1_, and is compared with the deviatoric stress-strain curve of the indoor experimental results, as shown in Figure 4. It can be seen that the macroscopic mechanical parameters of the GSR under two different working conditions are very similar to those of the indoor experiment. It was considered that the model was constructed correctly, so the simulation experiment of the other working conditions was carried out.

#### 2.2.2. Construction of the GSR

**Figure 5:**
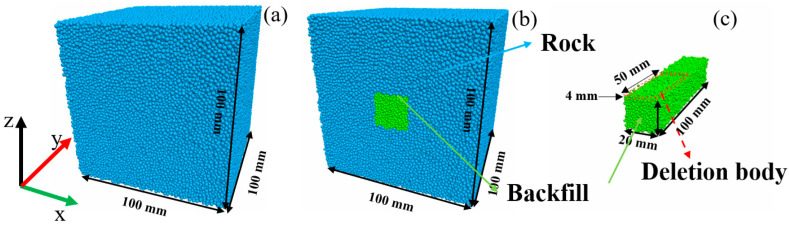
Model Construction (**a**) Rock samples; (**b**) GSR samples; (**c**) backfill.

The size of the GSR is 100 mm × 100 mm × 100 mm, so the pure rock model of 100 mm × 100 mm × 100 mm was constructed first, as shown in Figure 5a. According to the requirement of the aperture size, the middle part of the area was removed to generate the backfill in the area, as shown in Figure 5b. According to the different RCFR, a small part of the backfill was deleted, as shown in Figure 5c, and a small block of 50 mm × 20 mm × 4 mm was deleted to achieve a 50% RCFR effect. In this way, 25 models composed of five kinds of goaf sizes and five kinds of RCFR were established, and the number of particles per type was more than 200,000. 

#### 2.2.3. Arrangement of Measuring Ball

**Figure 6:**
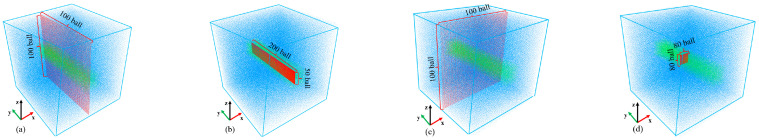
Arrangement of measuring balls (**a**) YZ plane; (**b**) YZ plane of backfill; (**c**) XZ plane; (**d**) XZ plane of backfill.

Firstly, the measuring ball was placed in the corresponding position, as shown in Figure 6. The quantity arrangement 100 × 100 ball, 80 × 80 ball, 200 × 50 ball, where Figure 6a,c is in the whole GSR and Figure 6b,d is in the backfill. These measuring balls were used to monitor the stress at the center of the GSR in real time. Finally, the z-direction stress detected in the measurement ball was stored in UDTensor and a stress cloud nephogram was generated.

## 3. Analysis of Experimental Results

The data obtained from the laboratory test in this section are shown in Table 2.

### 3.1. Stress-Strain Analysis

**Figure 7:**
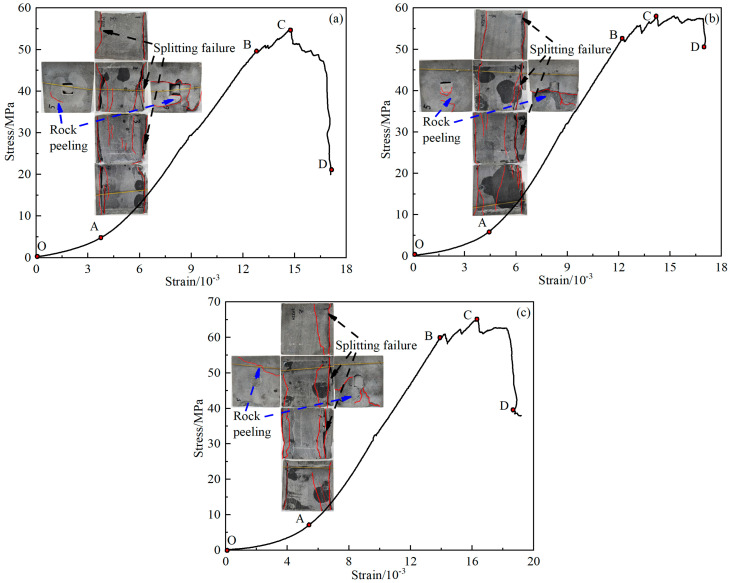
Stress-strain curve (**a**) GSR-20-0; (**b**) GSR-20-50; (**c**) GSR-20-100.

The stress-strain curve can express well the failure characteristics of the GSR during loading. Figure 7 shows the stress-strain curves and failure models of the GSR under different working conditions (this manuscript lists some GSR for analysis). By observing the stress-strain curve of the specimen during the biaxial loading compression experiment, the curve is divided into four stages.

1. Compaction stage (OA): The original cracks in the GSR are closed under load, while the backfill in the goaf is tightly connected to the rock wall under load compression, and the stress-strain curve is concave. Due to the tight connection stage between the backfill and the rock wall, the compression stage is long;
2. Elastic stage (AB): Different from the previous stage, the GSR sample has been compacted and a small number of cracks gradually appear inside. The stress and strain in this stage increase linearly;
3. Yield stage (BC): When the stress exceeds the yield limit, the particles can no longer be compressed, and their bond is damaged, resulting in a large number of cracks. As the loading continues, the cracks develop and spread in the weak area of the GSR, and the stress-strain curve fluctuates up and down;
4. Failure stage (CD): After the bearing capacity of the GSR reaches its peak, under the action of inter-particle friction, not all bearing capacity is immediately lost, and residual stress is clearly manifested. At this time, the GSR still has bearing capacity. The stress-strain curve at this stage rapidly decreases after a period of fluctuation.

From the failure characteristics, it can be seen that the failure behavior of the GSR is relatively simple. The main occurrence is splitting failure, which is longitudinally distributed on the surface of the sample, and large pieces of peeling usually occur on the left and right free surfaces, as shown in Figure 7.

The corresponding points of each stage on the stress-strain curve of the GSR were analyzed. Where *σ*_c_ corresponds to the point A stress, *σ*_y_ corresponds to the point B stress, and *σ*_p_ corresponds to the point C stress.

**Figure 8:**
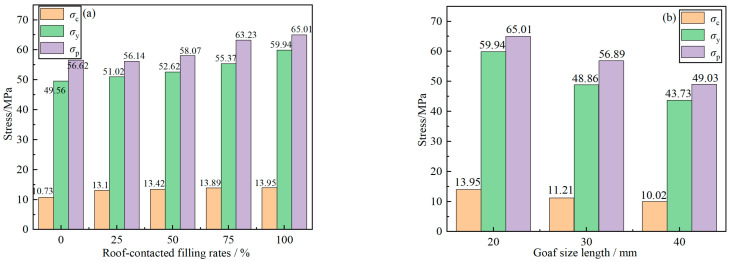
Stress histogram at each stage (**a**) The relationship between roof-contacted filling rate and strength; (**b**) The relationship between goaf size and strength.

As can be seen from Figure 8, when the goaf size is 20 mm, the RCFR increased from 0% to 100%. The stress of the three stages increased with the increase of the RCFR. The *σ*_c_ increased from 10.73 MPa to 13.95 MPa, with a growth rate of 30.0%. The *σ*_y_ increased from 49.56 MPa to 59.94 MPa, with a growth rate of 20.9%. The *σ*_p_ increased from 56.62 MPa to 65.01 MPa, with a growth rate of 14.8%. It can be seen from Figure 8b that when the goaf size increased from 20 mm to 40 mm, stress of the three stages decreased. The *σ*_c_ decreased from 13.95 MPa to 10.02 MPa, and the attenuation rate was 28.2%. The *σ*_y_ decreased from 59.94 MPa to 43.73 MPa, with an attenuation rate of 27.0%. The *σ*_p_ decreased from 65.01 MPa to 49.03 MPa, and the attenuation rate was 24.6%. It shows that the increase in the RCFR helps to improve the bearing capacity of the GSR, and the increase in the goaf size makes the strength of the sample decrease.

### 3.2. Analysis of Mechanical Characteristics of GSR

**Figure 9:**
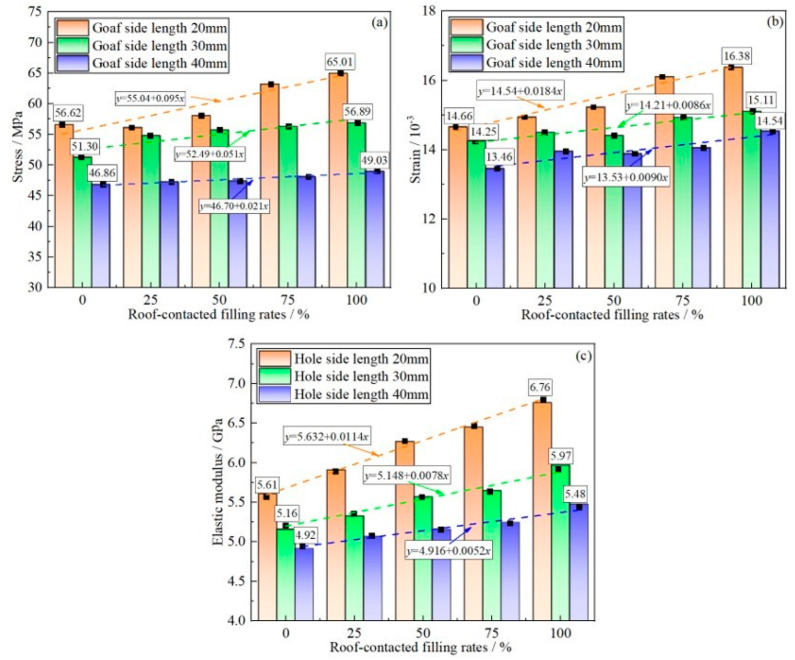
Mechanical variation characteristics (**a**) peak stress; (**b**) peak strain; (**c**) elastic modulus.

The peak stress, peak strain, and elastic modulus are the main mechanical parameters of the GSR. Figure 9 shows the relationship curves of peak stress, peak strain, and elastic modulus of each GSR with different goaf sizes and different RCFR.

As can be seen from Figure 9a, the peak strength of the GSR increases with the increase of the RCFR, and shows a good linear relationship, as shown in Table 3. The GSR samples with different goaf sizes have different growth rates when the RCFR increases from 0% to 100%. When the goaf size is 20 mm, the peak strength of the sample increases from 56.62 MPa to 65.01 MPa, with a growth rate of 14.8%. When the goaf size is 30 mm, the peak strength of the GSR increases from 51.30 MPa to 56.89 MPa, with a growth rate of 10.9%. When the goaf size is 40 mm, the peak strength of the sample increases from 46.86 MPa to 49.03 MPa, with a growth rate of 4.6%. It can be found that the increase in the goaf size makes the growth rate of the RCFR decrease the strength of the sample.

As can be seen from Figure 9b, the peak strain of the GSR increases with the increase of the RCFR, and shows a good linear relationship, as shown in Table 3, when the RCFR increases from 0% to 100%. The peak strain of the GSR increases from 14.66 to 16.38 when the goaf size is 20 mm, and the growth rate is 11.7%. When the goaf size is 30 mm, the peak strain of the sample increases from 14.25 to 15.11, with a growth rate of 6.0%. When the goaf size is 40 mm, the peak strain of the sample increases from 13.46 to 14.54, and the growth rate is 8.0%. It can be found that the increase of the goaf makes the growth rate of the RCFR decrease the strain of the sample.

As can be seen from Figure 9c, the elastic modulus of the GSR increases with the increase of the RCFR, and shows a good linear relationship, as shown in Table 3, when the RCFR increases from 0% to 100%. The elastic modulus of the GSR increases from 5.61 GPa to 6.76 GPa when the goaf size is 20 mm, and the growth rate is 20.5%. When the goaf size is 30 mm, the elastic modulus of the GSR increases from 5.16 GPa to 5.97 GPa, with a growth rate of 15.7%. When the goaf size is 40 mm, the elastic modulus of the GSR increases from 4.92 GPa to 5.48 GPa, with a growth rate of 11.4%. It can be found that the increase of the goaf makes the growth rate of the RCFR decrease the elastic modulus of the GSR.

The fitting formulas for the peak stress, peak strain, and elastic modulus of samples with different goaf sizes and RCFR are collected and listed in Table 3.

It can be seen from the table that the correlation is strong, indicating that the RCFR are closely related to the peak stress, peak strain, and elastic modulus of the GSR. As can be seen, with the increase in the size of the goaf, the peak strength, peak strain, and elastic modulus of the GSR decrease, while with the increase of the RCFR, the peak strength, peak strain, and elastic modulus of the GSR increase. The size of the goaf and the RCFR also affect each other; when the goaf size increases, the enhancement effect of the RCFR on the GSR becomes weaker.

### 3.3. Characteristic Analysis of Acoustic Emission Signal

The evolution law of rock microcracks under load—including closure, propagation, penetration, and complete failure—is described by the characteristics of acoustic emission signals. The elastic waves released during the crack change process are effectively monitored, and the internal deformation and failure of the rock are reflected to a certain extent. Ring counting is the number of oscillations of the transducer over the threshold signal, which is a monitoring datum related to signal amplitude in acoustic emission testing. It reflects the rock fracture situation and is widely used for analyzing the evolution of rock cracks [31,32,33,34,35,36,37].

**Figure 10:**
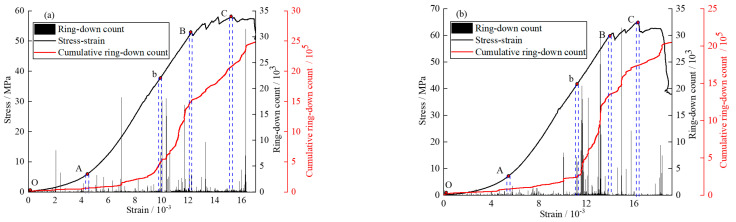
Acoustic emission ringing count signal distribution (**a**): GSR-20-50; (**b**): GSR-20-100.

In the elastic stage of the stress-strain curve, the acoustic emission signal characteristics are obviously different before and after the crack generation. Therefore, according to the characteristics of the acoustic emission signal, the sudden growth point is selected as the crack generation point in the elastic stage. As shown in Figure 10, point b is taken as the point before and after crack generation, and is divided into a no crack developed stage (Ab) and a crack developed stage (bB).

In the compaction stage (OA) and the no crack developed stage (Ab), the number of acoustic emission ringing count signals is small, most are less than 5000 times, and the cumulative acoustic emission ringing count is at a low level. In the crack developed stage (bB), the number of acoustic emission ringing count signals increases rapidly, and multiple acoustic emission ringing count peaks appear. The cumulative acoustic emission ringing count increases sharply; in Figure 10a, more than 15,000 times, and in Figure 10b, more than 20,000 times. In the yield stage (BC), the number of acoustic emissions ringing count signals is reduced compared with the bB stage, but there are still individual acoustic emission ringing count peaks, and the cumulative acoustic emission ringing count shows a “stepwise” growth. In the post-failure stage (CD), the number of acoustic emissions ringing count signals is greatly reduced, and the cumulative acoustic emission ringing count becomes a slow growth.

**Figure 11:**
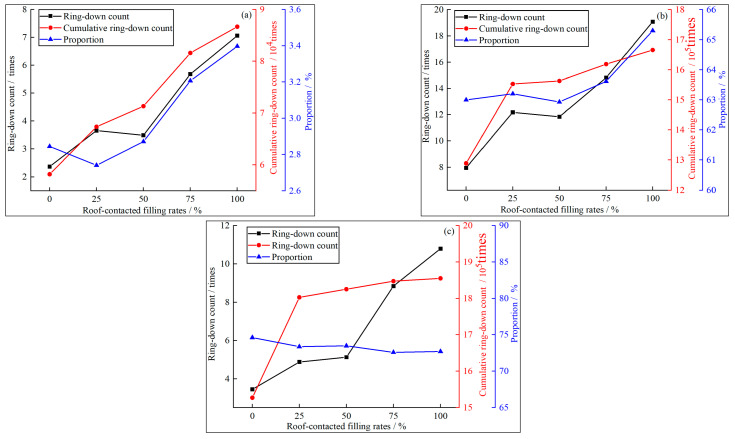
Ring-down count quantity relationship (**a**): Point A; (**b**): Point B; (**c**): Point C.

Quantitative analysis was conducted on the variation characteristics of acoustic emission ringing numbers with filling and capping rates at stage points A, B, and C. The average number of ringing numbers in the extremely small monitoring area before and after each stage point was taken, and a relationship diagram between acoustic emission ringing numbers and filling and capping rates was drawn based on the processed data, as shown in Figure 11. In the initial stage of loading, with the increase of the RCFR, the number of acoustic emission ring-down counts increased from 2 to 7 times, the cumulative acoustic emission ring-down counts increased from 58,000 to 87,000, and the total proportion of cumulative acoustic emission ring-down counts increased from 2.84% to 3.40%. It can be seen from Figure 11b that in the middle and late stages of loading, with the increase of the RCFR, the acoustic emission ring-down counts increased from 8 to 18 times, and the cumulative acoustic emission ring-down counts increased from 1.28 million to 1.67 million. The total proportion of cumulative acoustic emission ring-down counts increased from 60.2% to 65.3%. From Figure 11c, it can be seen that in the later stage of loading, with the increase of the RCFR, the acoustic emission ring-down counts increased from 4 times to 11 times, the cumulative acoustic emission ring-down counts increased from 1.53 million to 1.86 million, but the total proportion of cumulative acoustic emission ring-down counts reduced from 74.6% to 68.7%.

In summary, due to the increase in the RCFR, the peak stress and peak strain of the GSR sample increase, as do the signal intensity and duration of the acoustic emission ringing count. Therefore, the acoustic emission ringing count and the acoustic emission cumulative ringing count in the corresponding stage period increase with the increase of the RCFR. The increase in the RCFR can also slow down the damage speed of the GSR. Before the complete loss of bearing capacity, the acoustic emission ringing count signal is continuously generated inside the GSR. Therefore, the proportion of acoustic emission ringing counts in the post-peak period increases.

## 4. Crack Evolution in Combine Model

In indoor experiments, the generation and evolution process of internal cracks in the composite model cannot be directly observed, but this problem is solved through PFC numerical simulation. Therefore, this section will use PFC numerical simulation to study the crack evolution characteristics of the GSR during loading.

**Figure 12:**
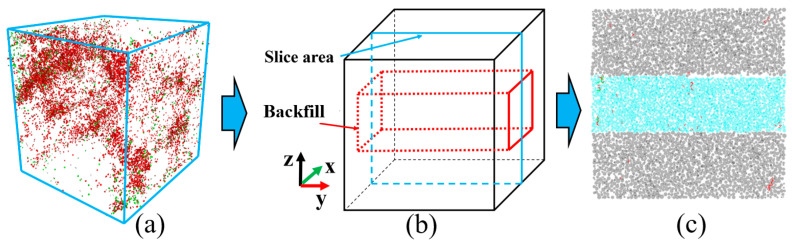
Slicing process (**a**) crack distribution of GSR; (**b**) slice position; (**c**) slice diagram.

**Figure 13:**
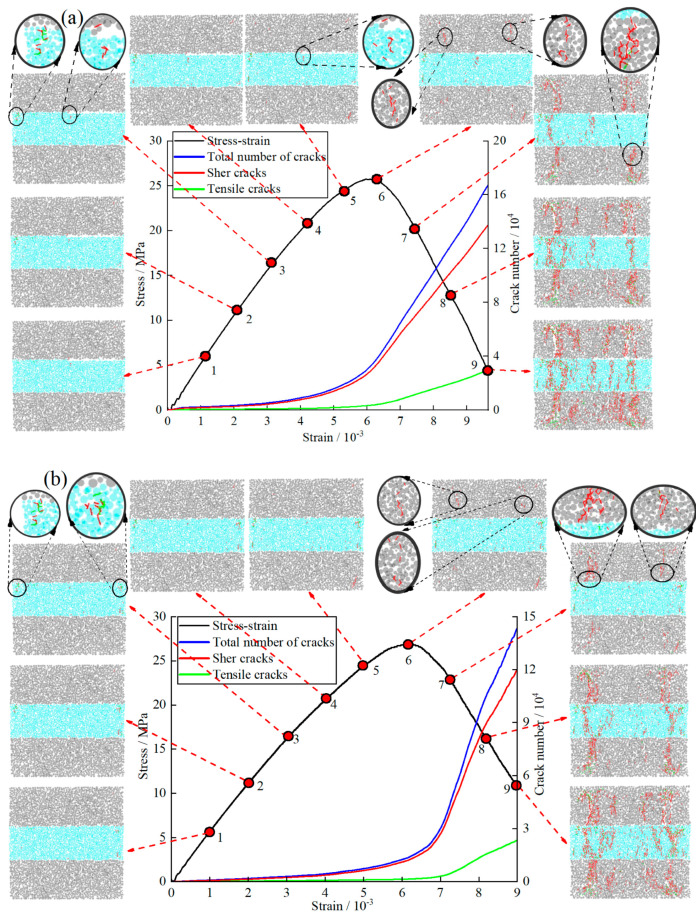
Crack evolution characteristics (**a**) GSR-30-50; (**b**) GSR-30-100; 1~9: Loading status every 10,000 steps.

The GSR was sliced, as shown in Figure 12. For the same GSR in the simulation process, the crack development diagram at each stage was obtained by slicing every 10,000 steps, and the relationship between the number of cracks and the strain was recorded, as shown in Figure 13. Figure 13a,b are GSR-30-50 and GSR-30-100, respectively.

As can be seen from Figure 13a, in the initial stage of loading (0–3), the internal stress of the GSR sample rarely exceeds the cohesion between the particles, and almost no cracks are produced. In the medium term of loading (3–6), cracks begin to appear in the GSR sample. The goaf is compressed, and the backfill in the roof-contacted part undergoes downward displacement under the pressure of the surrounding rock, while the backfill in the non roof-contacted part does not bear the pressure of the surrounding rock and does not undergo displacement. Therefore, shear cracks are prone to occur at the connection (marked in red). Under the compression of the rock mass, the end particles of the backfill soil are squeezed out, causing outward displacement, and tensile cracks (marked in green) are generated in the backfill. In the later stage of loading (6–9), the internal crack growth rate of the GSR sample rises sharply, and cracks are initiated at the left and right positions of the rock mass (red mark), and extend to the goaf position.

In the top filling area, the backfill is in direct contact with the rock mass, so when the goaf is damaged under pressure, the area is more likely to be damaged. Under the load, the crack growth rate of the rock mass is significantly faster than the backfill. The propagation speed of cracks in the rock mass is faster.

As can be seen from Figure 13b, in the medium term of loading (3–6), the crack generation rate in the GSR sample is slow. In the medium term of loading (3–6), tension cracks occur at both ends of the backfill in the sample (green mark). In the later stage of loading (6–9), there is crack initiation in the rock mass and extension to the goaf location. As the loading continues, the backfill accelerates the crack propagation under the pressure of the rock mass, and the cracks in the rock mass and the backfill are connected. Finally, the sample is spread all over the vertical crack zone, and the bearing capacity is lost.

**Figure 14:**
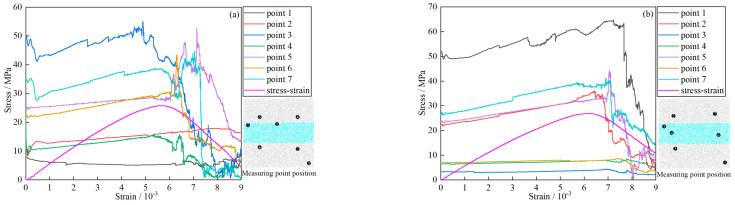
Stress-strain curve of measuring point (**a**) GSR-30-50; (**b)** GSR-30-100; 1~7: Crack development status.

In the crack evolution diagram in Figure 13, the initial crack initiation and propagation positions can be clearly obtained. The stress evolution at a certain position is monitored and recorded by the corresponding position measurement ball, and seven monitoring points are marked in the crack evolution nephogram. The location of the monitoring points is shown in Figure 14. Due to the influence of confining pressure, the initial position of the stress-strain curve in the figure at the measuring point is not 0.

As can be seen from Figure 14a, the peak stress of GSR-30-50 is 25.76 MPa. Before the peak stress of the GSR, the stress-strain curves of the measuring points are relatively gentle and maintain an upward trend with small fluctuations. When the GSR reaches the peak stress position, the whole sample tends to be in a state of instability and failure, and the stress at each point inside is continuously released, resulting up-and-down fluctuations in the stress-strain curve of the measuring point. At measuring points 3 and 5 inside the rock mass, the corresponding peak stress reaches 55.2 MPa and 52.9 MPa, which is more than twice the peak stress, indicating that stress concentration occurs at the measuring point. The peak stress of 6 and 7 reaches 43.4 MPa and 44.7 MPa, which is higher than the peak stress of the GSR. It shows that the stress concentration also occurs at the measuring point. However, compared with the stress concentration phenomenon at positions 3 and 5, the stress concentration phenomenon is weaker. The peak stress of measuring points 1 and 2 inside the backfill is 6.8 MPa and 18.0 MPa, which is lower than the peak stress of the GSR.

As can be seen from Figure 14b, the peak stress of GSR-30-100 is 26.89 MPa. Before the peak stress of the GSR, the stress-strain curves of the measuring points are relatively gentle and maintain an upward trend. When the GSR reaches the peak stress position, the stress-strain curve of the measuring point shows a significant up-and-down chaotic fluctuation. At measuring point 1 inside the rock mass, the peak stress reaches 64.7 MPa, which is 2.5 times the peak stress of the sample, indicating that stress concentration occurs at the measuring point. The peak stress of measuring points 2, 5, and 7 reaches 35.9 MPa, 44.4 MPa, and 41.2 MPa, which is higher than the peak stress of the GSR, indicating that the stress concentration also occurs at the measuring point. The peak stress of measuring points 3, 4, and 6 inside the backfill reaches 4.5 MPa, 7.7 MPa, and 8.7 MPa, which is lower than the peak stress of the GSR. This is because the mesoscopic parameters between the rock particles and the backfill particles are quite different, and the cohesion between the backfill particles is much smaller than that of the rock particles, so there is no large stress concentration in the backfill.

In PFC numerical simulation, the basic principles of “negative pressure and positive tension” forces are followed. The stress in the following stress nephogram is negative. The greater the negative value, the greater the pressure. This is not only consistent with the compression state of GSR, but also determines the location of the main stress concentration generated. The stress in the rock mass and backfill in GSR is studied and analyzed separately.

**Figure 15:**
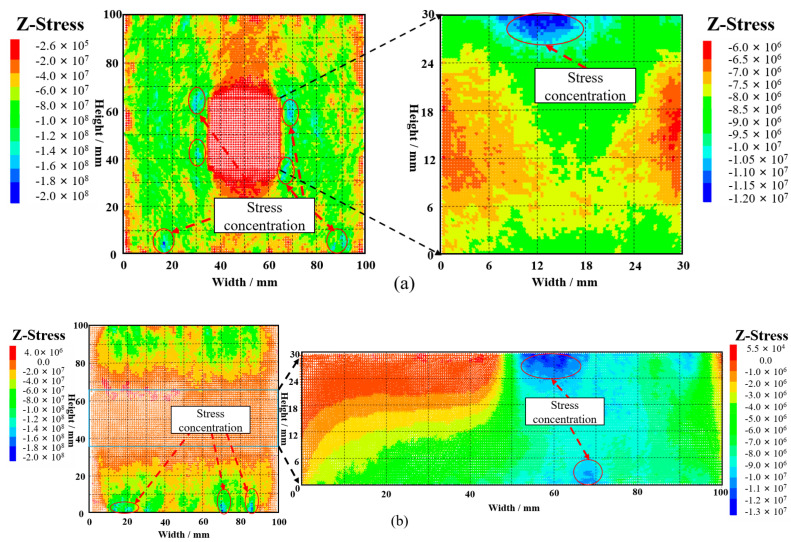
GSR-30-50 stress nephogram (**a**) xz plane; (**b**) yz plane.

Figure 15a shows the xz plane of the GSR-30-50 composite model. The stress concentration phenomenon of the rock mass occurs at the left and right positions at the bottom of the rock mass and at the four corners around the goaf. The stress concentration of the backfill occurs at the top. Figure 15b shows the yz plane of the GSR-30-50 composite model. The phenomenon of stress concentration occurs at the left and right positions at the bottom of the rock mass and near the top middle and bottom middle of the backfill.

**Figure 16:**
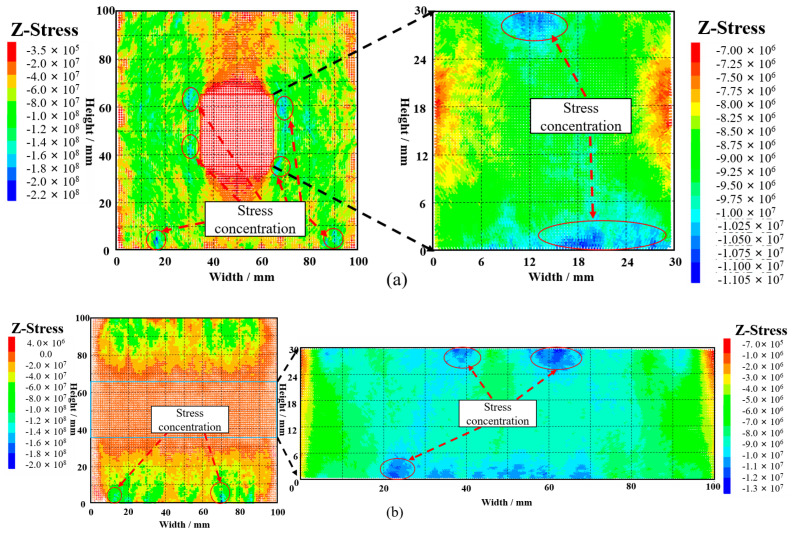
GSR-30-100 stress nephogram (**a**) xz plane; (**b**) yz plane.

Figure 16a shows the xz plane of the GSR-30-100 composite model. The phenomenon of stress concentration occurs at the left and right positions at the bottom of the rock mass and at the top and bottom of the backfill. Figure 16b shows the yz plane of the GSR-30-100 composite model. The stress concentration of the rock mass mainly occurs at the left and right positions at the bottom of the rock mass, The stress concentration of the backfill occurs in several areas at the top and bottom. Therefore, in the process of mining filling, it is necessary to improve the filling quality and ensure a high RCFR in order to improve the support effect of the backfill on the surrounding rock and ensure the safety and stability of the mining site.

Based on comprehensive analysis, the stress concentration phenomenon on the left and right sides of the goaf bottom and the top and bottom positions of the backfill in the GSR sample is prone to occur, causing the particle bonding at the positions to be destroyed and cracks to occur. This stress concentration phenomenon can be found in the stress cloud nephogram. As a stress release zone, the area where the backfill is not fully filled cannot directly participate in the load bearing capacity, which weakens the strength of the GSR composite specimen.

## 5. Conclusions

The real biaxial loading and numerical simulation experiments were carried out on the GSR with different goaf size and RCFR; simultaneously, acoustic emission monitoring was carried out. The main conclusions are as follows:

1. The stress and strain curve of the GSR can be divided into four stages: In the compaction stage, the primary crack and goaf are compressed, and the curve is concave. In the elastic stage, the particles are compressed and the curve rises linearly. In the yield stage, the bond between the microparticles is continuously destroyed, and the curve rises in fluctuation. In the failure stage, the crack gradually penetrates the sample and the curve drops sharply. The stress magnitude at each stage increases with the increase of RCFR, and decreases with the increase of goaf size;
2. The peak stress, peak strain, and elastic modulus of GSR increase linearly with the increase of RCFR, and decrease with the increase of goaf size. When the RCFR increases from 0% to 100%, the peak stress, peak strain, and elastic modulus of the GSR sample with a goaf size of 20 mm have the highest growth amplitude, with 14.8%, 11.7%, and 20.5%, respectively;
3. The acoustic emission ringing count of the GSR sample significantly increases during the bC stage, and multiple ringing count peaks appear. The cumulative ringing count curve shows a “stepped” growth. The acoustic emission ringing count at each stage point increases with the increase of RCFR, but the proportion of acoustic emission ringing count at peak stage point C decreases with the increase of RCFR;
4. During the loading process of the GSR samples, shear failure mainly occurs, and the initiated failure cracks are generated from the left and right ends of the filling joint between the rock mass and the backfill. According to the growth of RCFR, the crack initiation position changes accordingly. The rock mass in the GSR sample is prone to shear cracks, while the filling material is prone to tensile cracks, and the number of cracks increases faster in the rock mass;
5. In the GSR sample, stress concentration is prone to occur at the crack initiation site, and failure occurs during the peak stage to release stress. The peak concentration stress in the rock mass and backfill is 1–2.5 times and 0.17–0.7 times the GSR peak stress, respectively. From the stress cloud nephogram, it can be seen that the four corners of the goaf and the middle of the backfill in the GSR sample are subject to stress concentration.

## Figures and Tables

**Table 1:** GSR Micro-mechanical parameters of PFC3D.

Micro-Parameters	Rock	Backfill	Rock-Backfill
Minimum radius of the particle R_min_ (mm)	0.8	0.64	-
Maximum radius of the particle R_max_ (mm)	1.2	0.96	-
Density of the particle, *ρ* (kg/m^3^)	2500	1700	-
Friction coefficient, *μ*	0.7	0.7	0.7
Normal-to-shear stiffness ratio, *k*_n_/*k*_s_	2	2	2
Linear Bond effective modulus *E* (GPa)	8.44	0.15	0.175
Linear Parallel Bond effective modulus *E* (GPa)	8.44	0.15	0.175
Linear Parallel Bond Tensile strength *σ*_c_ (MPa)	50.02	3.9	4.2
Linear Parallel Bond Cohesion *c* (MPa)	25.01	1.95	2.1
Friction angle *ϕ* (°)	45	30	30

**Table 2:** Experimental result of biaxial loading of the GSR.

Specimen Number	Peak Stress *σ*_1_/MPa	Peak Strain *ε*_1_/10^−3^	Elastic Modulus *E*_0_/GPa
GSR-20-0	56.62	14.66	5.61
GSR-20-25	56.14	14.94	5.91
GSR-20-50	58.07	15.23	6.27
GSR-20-75	63.23	16.11	6.45
GSR-20-100	65.01	16.38	6.76
GSR-30-0	51.30	14.25	5.16
GSR-30-25	54.84	14.50	5.33
GSR-30-50	55.76	14.41	5.57
GSR-30-75	56.31	14.93	5.65
GSR-30-100	56.89	15.11	5.97
GSR-40-0	46.86	13.46	4.92
GSR-40-25	47.27	13.96	5.07
GSR-40-50	47.42	13.89	5.16
GSR-40-75	48.11	14.06	5.25
GSR-40-100	49.03	14.54	5.48

Annotation: GSR-20-0 represents backfill goaf surrounding rock; the goaf size is 20 mm, RCFR is 0%, and so on.

**Table 3:** Fitting formulas of peak stress, peak strain, and elastic modulus of the GSR.

Fitting Formulas	Goaf Size/mm	Fitting Formulas	R2
Peak stress	20	y = 55.04 + 0.095x	0.8711
	30	y = 52.49 + 0.051x	0.8173
	40	y = 46.70 + 0.021x	0.9257
Peak strain	20	y = 14.54 + 0.0184x	0.9521
	30	y = 14.21 + 0.0086x	0.8728
	40	y = 13.53 + 0.0090x	0.8528
Elastic modulus	20	y = 5.632 + 0.0114x	0.9918
	30	y = 5.148 + 0.0078x	0.9742
	40	y = 4.916 + 0.0052x	0.9662

## Data Availability

No new data were created or analyzed in this study. Data sharing is not applicable to this article.
